# Investigation on Seismic Behavior of a Novel Precast Shear Wall System with Different Infill Wall Constructions

**DOI:** 10.3390/ma16237343

**Published:** 2023-11-25

**Authors:** Min Sun, Sheng Zhang, Jun Yang, Youzhen Fang, Xiaochun Xu

**Affiliations:** 1 Jiangsu Key Laboratory of Structure Engineering, Suzhou University of Science and Technology, Suzhou 215011, China; sunmin@usts.edu.cn (M.S.); ultralifezs@163.com (S.Z.); fyz72@mail.usts.edu.cn (Y.F.); 2 Suzhou Wujiachen Construction Industry Co., Ltd., Suzhou 215011, China; 13913525799@163.com

**Keywords:** precast shear wall, infill wall, seismic behavior, angle steel connector, full scale, cyclic loading test

## Abstract

Construction industrialization addresses various challenges in the traditional construction industry, enabling building structures to conserve resources and enhance energy efficiency while reducing emissions. Precast shear walls involve the factory-based production of components, followed by transportation to a construction site for assembly. The method of connecting these components is crucial for precast concrete shear wall systems. Common connection methods include lap-spliced connections, post-tensioned connections, welded connections, bolted connections, and sleeve connections. However, challenges such as construction precision and technology proficiency have limited their application. In response, a novel precast concrete shear wall system utilizing angle steel connectors has been proposed. These angle steel connectors enhance the shear resistance of horizontal joints between precast concrete shear walls and the foundation, providing provisional support for specimen positioning and installation. Presently, the seismic performance of this innovative precast shear wall system under the combined actions of cyclic horizontal loads and axial pressure or tension has been extensively investigated. In practical engineering applications, precast concrete shear wall systems are often accompanied by infill walls. However, there is limited research on the seismic performance of precast concrete shear wall systems with infill walls. To address this gap, this study designed and fabricated two novel precast concrete shear walls with different infill wall constructions. One specimen featured an infill wall composed of a single wall panel, while the other had an infill wall consisting of two panels. Pseudo-static tests were conducted on both specimens under constant axial compression. Subsequently, the seismic performance and force mechanism of the two specimens were compared with the novel precast concrete shear walls without infill walls. The test results demonstrated that the specimen with two infill wall panels exhibited superior overall performance compared to the one with a single continuous infill wall panel. Furthermore, it was observed that, during the loading process, the edge columns of specimens with infill walls provided the majority of the increased load-bearing capacity, while the infill walls made a limited contribution to the overall load-bearing capacity of the structures.

## 1. Introduction

With the rapid development of the global economy and the concurrent surge in productivity, a multitude of challenges, including resource depletion, environmental contamination, and quality disparities, have emerged. As imperatives for development, energy conservation, emission reductions, and environmental protection have gained paramount importance. Construction industrialization can address many problems in the traditional construction industry. It empowers structures to preserve resources and curtail energy consumption, effectively enhancing energy efficiency and minimizing emissions. Furthermore, in the realm of environmental stewardship, it plays a vital role in curtailing the generation of construction waste, mitigating construction-related noise, and reducing wastewater discharge. [1,2,3,4,5]. The seismic considerations for dynamic loads should align with the specific region where the intended application is located [6,7]. Güllü et al. [8,9] employed various numerical and finite element models to simulate errors, with these models representing different dynamic effects and the practical application area of Fourier spectrum calculations. At present, shear wall systems integrated into building structures exhibit noteworthy lateral stiffness, adeptly meeting the structural demands of high-rise buildings, and have found widespread application in precast concrete structure systems [10,11,12].

Precast shear walls involve the factory-based production of various components, which are subsequently transported to the construction site for assembly. The method used to connect these components constitutes a central concern for precast shear wall systems. The reliability of the connection method is pivotal in establishing effective force transmission mechanisms and ensuring overall structural integrity [13,14,15,16]. A literature review indicates that the prevalent methods for connecting precast concrete shear wall systems presently encompass lap-spliced connection [17,18], post-tensioned connection [14,19,20], welded connection, bolted connection [16,21,22], and sleeve connection [23,24,25].

Feng et al. [17] explored a novel connection method for upper and lower precast shear walls where the vertical reinforcement bars in the lower precast shear walls were bundled and extended into preformed holes in the upper precast shear wall. The results indicate that the proposed precast shear walls exhibit seismic performance comparable to or even exceeding that of cast-in-place shear walls, featuring a full hysteresis curve. Li et al. [18] studied the shear performance of vertical joints with different lapping splices in precast concrete frame–shear wall structures. Chalarca et al. [14] and Kurama et al. [19] investigated the behavior and seismic performance assessment of unbonded post-tensioned precast concrete walls, revealing that structural systems based on such walls demonstrated reliable seismic performance. Tiwari et al. [20] provided a detailed review of the experimental investigation of post-tensioned shear walls undertaken in the past. Also, they discussed different types of energy dissipators used in the past to improve the energy dissipation capability of post-tensioned shear walls. Mi et al. [16] introduced a precast tolerance concrete shear wall (PTCW) characterized by proper-length steel connectors. Their findings emphasized that the use of steel connectors to reinforce joints aligns with the seismic design concept of strong joints and weak members. Sun et al. [21] introduced an innovative high-strength bolted connection to address the problem of stress concentration in concrete. Zhao et al. [22] proposed a simplified tensile–bolt connection and a shear bolt–steel plate connection for low-rise, wall-based structures in earthquake-prone zones, which could adapt to the fast, easy, and reliable installation manners on construction sites. Wu et al. [23] explored the seismic performance of a precast short-leg shear wall using a grouting sleeve connection. Testing results indicated the reliability of the reinforcement bars connected by grouting sleeves before yielding. Wei et al. [24] conducted an experiment to investigate the seismic behavior of a precast RC frame–shear wall structure using full/half grout sleeve connections. The study concluded that the RC frame–shear wall structure exhibited commendable seismic performance. Zhou et al. [25] proposed a confined prestressed hollow core wall panel that utilizes connection reinforcement, partial grouting, and sleeve connections to link precast components. Some scholars have proposed increasing the seismic performance and comfort of prefabricated structures by installing structural bracings and energy dissipation devices [26,27]. Previous research [13,14,15,16,17,18,19,20,21,22,23,24,25,26,27] has demonstrated that, given reliable design and construction practices, common methods for connecting precast concrete shear wall systems can achieve commendable seismic performance.

**Figure 1:**
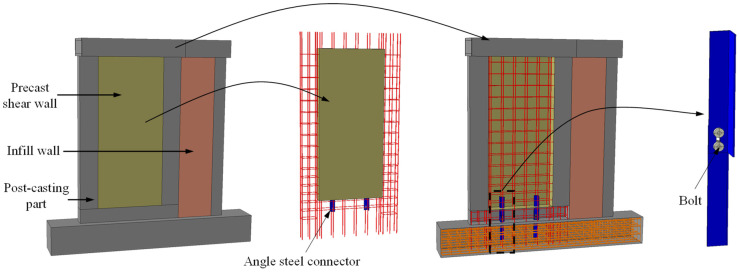
Configuration of the novel precast shear wall system with angle steel connectors.

However, practical construction often encounters challenges stemming from management issues or worker skill limitations, leading to occasional construction defects, such as grouting issues. These defects can significantly compromise structural performance. Some scholars have investigated the impact of grouting defects on the seismic performance of precast concrete shear walls, finding that they could lead to substantial degradation in structural seismic performance and even alter the structural failure mode [28,29]. Welding, bolting, prestressing, and similar techniques demand a high degree of construction precision and technological expertise, which has restricted their application in connecting precast concrete shear walls. To this end, Yang et al. introduced an innovative precast concrete shear wall system featuring angle steel connectors [10], as illustrated in Figure 1. This new system was designed based on a precast concrete shear wall with lap-spliced connections. Angle steel connectors are strategically deployed to enhance the shear resistance at horizontal joints between precast concrete shear walls and the foundation. They also offer provisional support for specimen positioning and installation. These components collectively ensure a secure connection of the precast concrete wall system, achieved through the integration of angle steel connectors and concrete pouring in the connection areas. This new precast shear wall system with angle steel connectors has been applied in a residential district of commercial housing in Suzhou, China. Through calculations, it has been determined that under equivalent design conditions, its cost was lower than that of shear wall structures using grouting sleeve connections.

**Figure 2:**
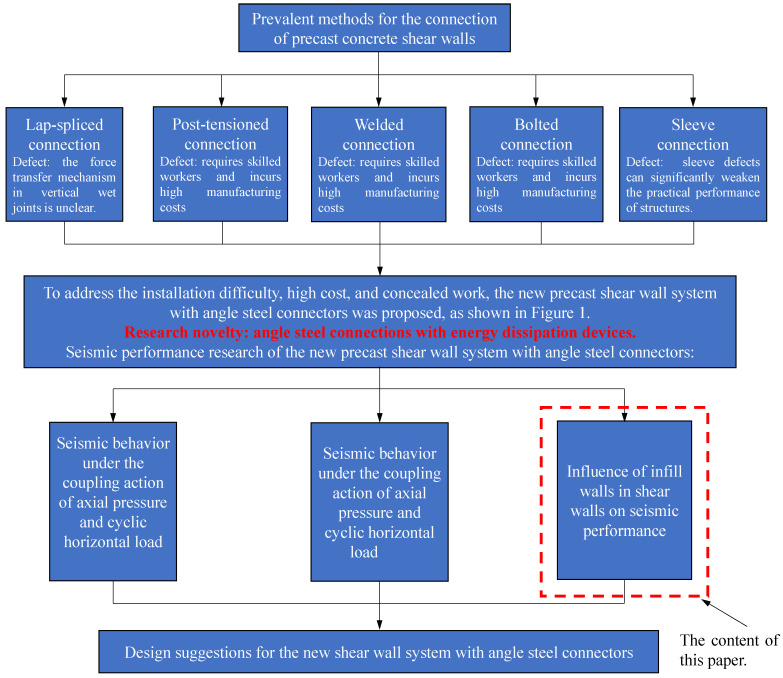
Research framework [10,30].

Yang et al. have investigated the seismic performance of the novel precast shear wall system subjected to the coupling action of cyclic horizontal load and axial pressure or axial tension [10,30]. According to the test results, the connections in the novel precast shear wall system exhibited high reliability, demonstrating significantly superior performance compared to monolithic cast-in-place connections. In practical engineering applications, precast concrete shear wall systems are often accompanied by infill walls. The infill walls, serving as the primary nonstructural components, exert a significant influence on the seismic behavior of main structures during seismic events [31]. However, limited research has been conducted on the seismic behavior of precast concrete shear wall systems with infill walls. To this end, this study designed and fabricated two novel precast concrete shear walls with infill walls. One specimen had an infill wall composed of a single wall panel, while the other specimen had an infill wall composed of two wall panels. The seismic testing methods in the laboratory include pseudo-static test methods, pseudo-dynamic test methods, and seismic simulation shake table test methods. Additionally, seismic fragility analysis methods and seismic risk mitigation and management techniques were employed to evaluate the seismic performance of precast concrete structures [32,33]. This study focused on investigating the contribution of shear walls and infill walls to the seismic performance of the new precast shear wall system, as well as the impact of infill wall construction on structural performance. In line with the comprehensive research objectives, this study utilizes the pseudo-static test method to conduct relevant experimental research. This study conducted pseudo-static tests on the specimens under constant axial compression and subsequently compared their seismic performance and force mechanisms with the novel precast concrete shear walls without infill walls. The objective was to provide valuable insights for the design and construction of precast concrete shear walls. Figure 2 shows the research framework diagram and research objective of this paper.

## 2. Description of Experiments

### 2.1. Specimen Design

**Figure 3:**
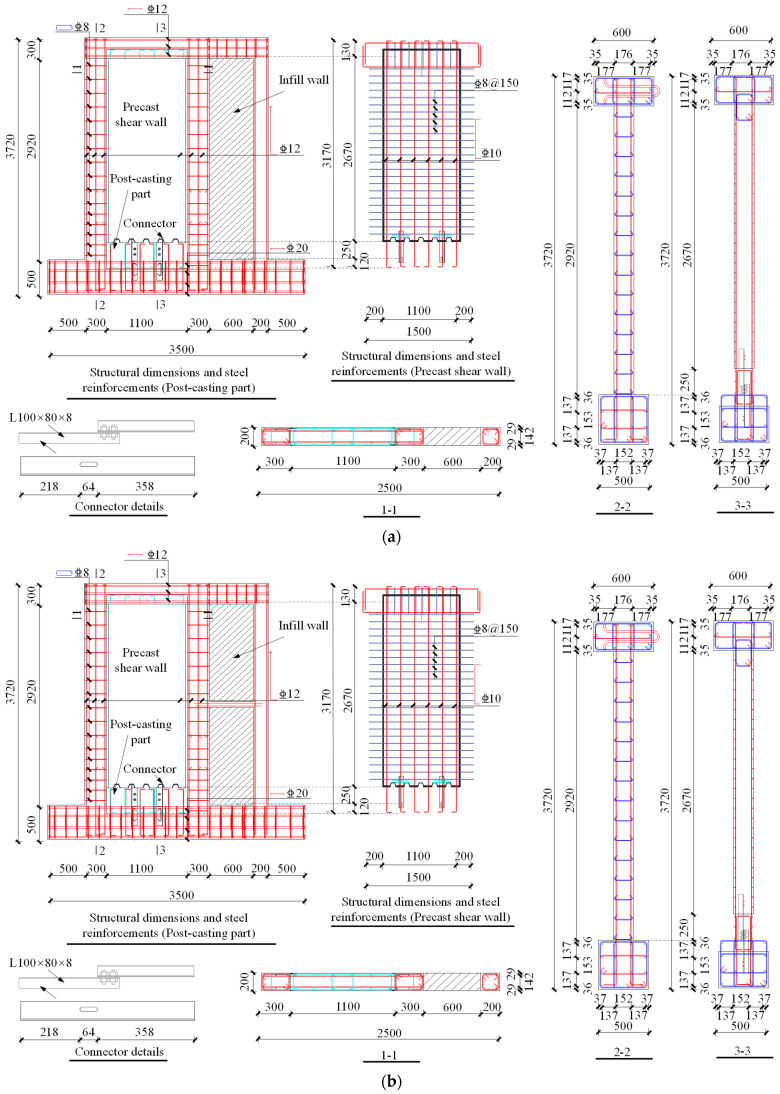
Structural dimensions and steel reinforcements of the specimens (unit: mm; 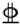: HRB400): (**a**) specimen 1 and (**b**) specimen 2.

In accordance with the guidelines outlined in Chinese codes [34,35,36], two full-scale specimens of the novel precast shear wall system were designed and fabricated, designated as specimen 1 and specimen 2. The concrete used was of grade C40, and the reinforcing steel bars were of HRB400 grade. The infill wall was constructed using aerated concrete blocks. The angle steel connectors were forged from Q235 unequal angle steel with M16-grade bolts. Specimens 1 and 2 measured 2920 mm (height) × 1700 mm (width) × 200 mm (thickness). The loading beam had a cross-sectional size of 300 mm × 600 mm, while the foundation beam measured 500 mm × 500 mm. The infill wall of specimen 1 comprised a single complete panel measuring 2920 mm × 600 mm × 200 mm, while the infill wall of specimen 2 comprised two panels, each measuring 1420 mm × 600 mm × 200 mm. Detailed structural dimensions and steel reinforcements are provided in Figure 3. For reference, our research team had previously conducted experiments on the seismic performance of the novel precast shear wall system without infill walls [10], referred to as specimen 3 in this paper. Additionally, this paper presents a comparative analysis of the data obtained from specimens 1 and 2 with that of specimen 3.

### 2.2. Material Mechanical Properties

During the pouring process, five standard cubic blocks with dimensions of 150 mm × 150 mm × 150 mm were prepared for each specimen. These blocks were then cured under identical conditions as the specimens for a period of 28 days. The average standard value of the concrete cube compressive strength, measured in accordance with the standards outlined in reference [37], is presented in Table 1. For the filling walls, aerated concrete blocks with an average compressive strength of 9.8 MPa were used. The mechanical properties of the steel were evaluated as described in reference [38], and the results are summarized in Table 2.

### 2.3. Load Protocol and Test Setup

**Figure 4:**
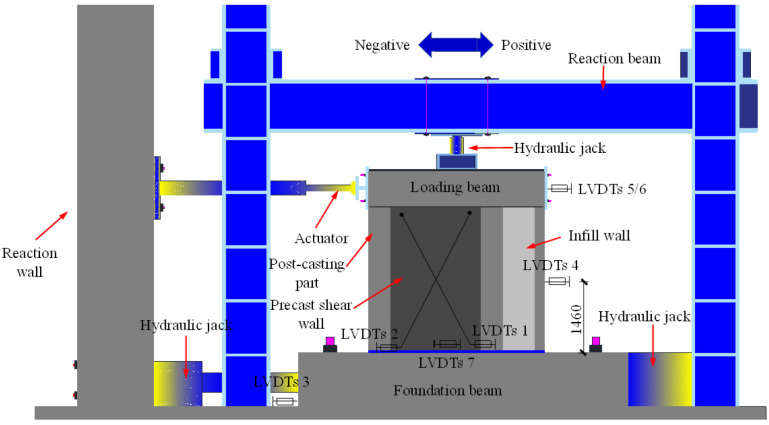
Test setups.

**Figure 5:**
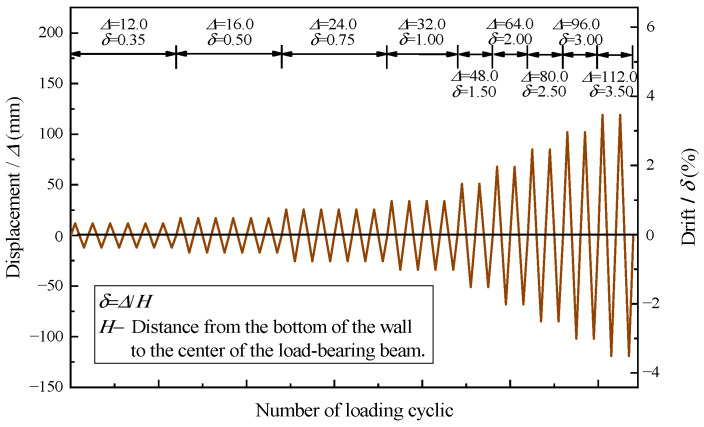
Displacement−controlled test sequence.

The schematic diagram of the test setups is shown in Figure 4. A 2500 kN class hydraulic jack was employed to apply vertical axial loading, and it was securely fixed on the sliding track. To ensure uniform force transmission, steel plates were embedded on the top surface of the loading beams. The horizontal cyclic load device consisted of an electro-hydraulic servo actuator, a reaction wall, and a horizontal connecting device. One end of the device was connected to the center of the loading beam end via the actuator axis, while the other end was anchored on the reaction wall. To prevent out-of-plane torsion of the specimens, an out-of-plane support, comprising a steel frame and steel beam, was provided for the loading beams. The foundation beams of the specimens were constrained both horizontally and vertically by steel pressure beams, ground anchor bolts, and jacks. The experimental loading process consisted of two steps. Initially, a constant axial pressure of 480 kN (resulting in an axial pressure ratio of 0.1) was applied at the top of the specimens. Subsequently, a 5 mm preloading was executed and cycled once to verify the proper functioning of all measurement points and testing equipment. Upon successful verification, the formal experimental loading commenced. The experiment employed a displacement control method, with lateral displacement set at 0.35%, 0.50%, 0.75%, and 1.00% for each stage. It then increased by 0.5% in subsequent stages until the horizontal bearing capacity of the specimen decreased to below 85% of the peak value or the horizontal displacement exceeded the limit of major seismic displacement. The corresponding amplitudes for each stage were 12 mm (0.35%, cycled 6 times), 16 mm (0.50%, cycled 6 times), 24 mm (0.75%, cycled 6 times), 32 mm (1.00%, cycled 4 times), 48 mm (1.50%, cycled 3 times), 64 mm (2.0%, cycled 3 times), 80 mm (2.5%, cycled 3 times), 96 mm (3.0%, cycled 2 times), and 112 mm (3.5%, cycled 2 times). Figure 5 shows the loading process for the specimens.

## 3. Results and Discussion

### 3.1. Damage Progression and Failure Modes

**Figure 6:**
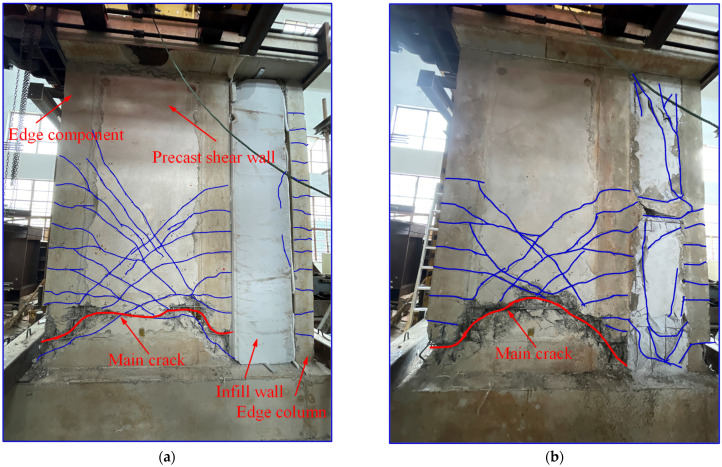
Failure modes of the specimens: (**a**) specimen 1 and (**b**) specimen 2.

Figure 6a shows the failure mode of specimen 1. When the horizontal displacement of the specimen reached 16 mm (*δ* = 0.5%), multiple horizontal cracks appeared on both sides of the specimen, and there were more cracks on the left side of the specimen. On the edge column, there also appeared multiple cracks, similar in height to those observed on the wall. As the horizontal displacement increased to 32 mm (*δ* = 1.0%), cracks in the specimen developed rapidly. A multitude of cracks appeared in the lower half of the specimen, while the horizontal cracks originating from the edge components extended towards the center of the shear wall, gradually transitioning into diagonal cracks. Some of these cracks extended from the horizontal cracks in the edge components to the middle of the wall, eventually evolving into a horizontal crack spanning the entire shear wall. Notably, no discernible cracks were evident in the infill wall section at this point. Upon reaching a horizontal displacement of 48 mm (*δ* = 1.5%), the specimen reached its peak load-bearing capacity, resulting in a deceleration of crack propagation. X−shaped cracks, inclined at a 45° angle, emerged on both sides of the specimen, with similar heights on each side. The horizontal cracks in the edge column extended to a considerable height, while no conspicuous cracks were observed in the infill wall section. When the horizontal displacement of the specimen reached 80 mm (*δ* = 2.5%), cracks fully developed, and a minor amount of concrete dislodged within a range of 200 mm to 400 mm from the base of the shear wall. When the horizontal displacement of the specimen reached 96 mm (*δ* = 3%), the load-bearing capacity dropped to below 85% of its peak value. The cracks in the infill wall section were less pronounced, but those in the edge column of the infill wall extended to the top of the specimen. Within the range of 0 mm to 600 mm from the foundation beam, the concrete at the corner positions of the specimen and the lower corner positions of the precast shear wall exhibited arch-shaped compressive damage. Additionally, the stirrups exhibited outward bulging, and the longitudinal reinforcement experienced buckling deformation, ultimately culminating in the failure of the specimen.

Figure 6b shows the failure mode of specimen 2. As the horizontal displacement of the specimen reached 16 mm (*δ* = 0.5%), multiple horizontal cracks appeared on the left side of the specimen. Furthermore, numerous horizontal cracks emerged on the edge column, mirroring those on the left side in terms of height. Upon reaching a horizontal displacement of 32 mm (*δ* = 1.0%), the crack propagation rate increased. A large number of cracks appeared in the lower section of the specimen, originating from the horizontal cracks in the edge components and extending towards the center of the shear wall. These progressed into diagonal cracks, paralleled by similar horizontal and diagonal cracks in the infill wall section. At a horizontal displacement of 48 mm (*δ* = 1.5%), the specimen reached its peak load-bearing capacity. The rate of crack expansion gradually decelerated. Diagonal cracks, inclined at a 45° angle, formed on both sides of the specimen, forming an X-shaped pattern. The heights of the cracks on both sides were roughly equivalent. The horizontal crack development height in the edge column was similar to that in the shear wall, and there was masonry detachment at the corner of the infill wall. When the horizontal displacement of the specimen reached 80 mm (*δ* = 2.5%), cracks developed extensively. The protective layer beside the cracks fell off. Diagonal cracks inclined at a greater angle as they extended upwards. They extended toward the corner of the shear wall, with a small amount of concrete detachment occurring within 0–300 mm from the bottom of the shear wall. Masonry detachment occurred at the corner and top of the infill wall. At a horizontal displacement of 96 mm (*δ* = l 3%), the load-bearing capacity dropped below 85% of its peak value. The entire masonry of the infill wall section fell off. The crack development height in the edge column was roughly equivalent to the crack height in the shear wall. Within 0−600 mm from the foundation beam, the concrete at the corner positions of the components and the lower corner positions of the prefabricated wall were crushed, with exposed steel reinforcement at the detachment location. Stirrups bulged out, and longitudinal reinforcement buckled. The severely damaged section exhibited a shape similar to an arch. As illustrated in Figure 6, the specimens exhibited an ultimate failure mode characterized by flexural failure, evident through the presence of a prominent horizontal main crack. As previously discussed, this mode of failure was classified as ductile, demonstrating a distinct progression during its development.

Upon comparing the failure modes of specimens 1 and 2, it was evident that both exhibited a common trait of flexural–shear failure in the wall sections. This was characterized by horizontal cracks propagating from edge elements towards the central wall, forming an X-shaped diagonal crack pattern. Upon failure of the specimens, the concrete at the base of the wall displayed arching compression failure, coupled with yielding and outward bulging of the reinforcements. The distinguishing factor lay in the behavior of specimen 2, where concrete spalling occurred in the corners of the infill walls and around connecting beams. In contrast, specimen 1 exhibited fewer cracks in the infill wall section, with notable cracks observed mainly in the edge columns. Additionally, specimen 2 displayed a more extensive development of cracks within the shear wall section compared to specimen 1. Experimental observations indicated that specimen 2 demonstrated a more comprehensive progression of plastic damage, showcasing superior overall structural integrity when compared to specimen 1.

### 3.2. Hysteresis Characteristics

**Figure 7:**
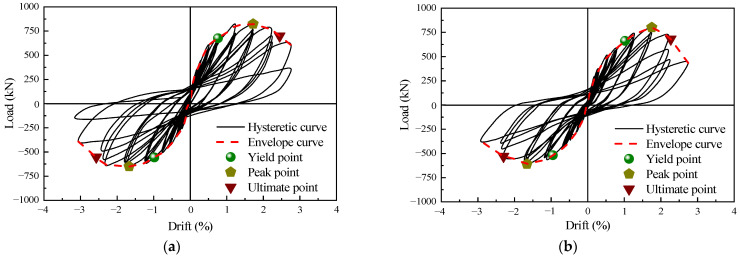
Horizontal load–displacement/drift hysteresis curves: (**a**) specimen 1 and (**b**) specimen 2.

Hysteresis curves directly quantify the relationship between force and deformation of specimens under the combined influence of axial force and cyclic horizontal loads. They also serve as crucial indicators of seismic performance, encompassing load-bearing capacity and energy dissipation. The experimental data were comprehensively processed, and hysteresis curves for each specimen were obtained through calculations, as shown in Figure 7.

Specimen 1 was equipped with a complete infill wall panel. From an overall perspective, the hysteresis curve was relatively full. Simultaneously, specimen 1 showed a higher load-carrying capacity compared to specimen 3 [10]. In the initial stages of the loading process, horizontal cracks appeared on both sides, resulting in residual deformation. The hysteresis curve area was extremely small, indicating minimal energy dissipation. When loaded to a lateral drift of 1.0%, cracks in the lower part of the specimen developed rapidly. Multiple horizontal cracks extended towards the middle of the wall, and the edge column gradually developed new cracks upwards. Residual deformation and energy dissipation increased gradually, and the hysteresis curve exhibited a certain degree of pinching. When loaded to a lateral drift of 1.5%, the load-carrying capacity of specimen 1 reached its peak value. Horizontal cracks extended and gradually formed diagonal cracks. The diagonal cracks on both sides took on an X-shaped pattern, and the hysteresis curve exhibited a spindle-shaped configuration. Continuing to load to 2.0% lateral drift, the diagonal cracks extended towards the corners of the wall, and a small amount of concrete at the wall corner was crushed. The rate of increase in the hysteresis loop area was significant. Further loading led to an arch-shaped collapse of the concrete, resulting in a markedly increased hysteresis area. Eventually, the ultimate load dropped to 75% of its peak load, marking the end of the loading process.

The hysteresis curve of specimen 2 exhibited similar characteristics to that of specimen 1. In the initial stages of the loading process, there were minor horizontal cracks observed at the edge column. The specimen was in the elastic phase, and the hysteresis curve was approximately linear. As loading continued, horizontal cracks appeared in the lower part of the edge components of the wall. The area of the hysteresis curve increased. When the lateral drift reached 1.0%, the crack propagation speed accelerated. Horizontal cracks formed in the edge components extended towards the middle of the wall, causing a certain degree of pinching in the hysteresis curve. The rate of increase in the hysteresis curve area was rapid. When the lateral drift reached 1.5%, the load-bearing capacity of the specimen reached its peak value. The cracks in the precast shear wall developed into diagonal cracks, and cracks in the infill wall propagated rapidly. The residual deformation of the specimen increased, and the masonry at the corner of the infill wall fell off. When the lateral drift reached 2.0%, the corner section in the lower part of the precast shear wall, which had suffered severe damage, exhibited an arch-like shape. As loading continued, the stiffness of the specimen degraded, exhibiting relative sliding. The load-bearing capacity decreased significantly, and longitudinal reinforcement buckling deformation occurred. The hysteresis curve area continued to increase, and the ultimate bearing capacity decreased to 66% of the peak load before the loading concluded.

Compared to specimen 1, specimen 2 exhibited a greater number of cracks and more severe damage in the infill wall panels. Furthermore, the hysteresis curve of specimen 2 demonstrated a fuller response, indicating a more comprehensive development of plastic damage. These observations suggested that specimen 2 demonstrated superior overall performance and energy dissipation capacity.

### 3.3. Load-Bearing Capacity and Characteristic Drift

**Figure 8:**
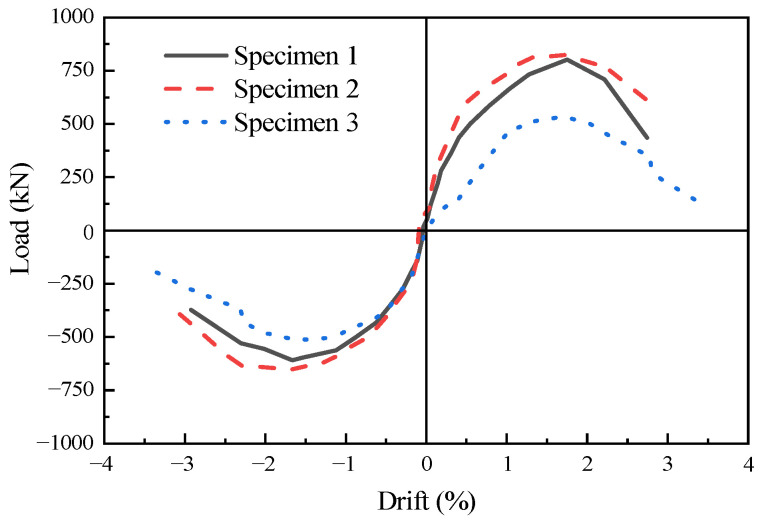
Comparison of the skeleton curves of the specimens [10].

Table 3 shows the characteristic values of the load-bearing capacity of the specimens, and Figure 8 shows the comparison of the skeleton curves of the specimens. Specimen 1 and specimen 2, due to differences in the construction of the infill wall section, exhibited similar trends in their skeleton curves. However, specimen 2 had a higher initial stiffness. The positive yield load of specimen 2 (677.98 kN) was 1.02 times that of specimen 1 (662.13 kN). The negative yield load of specimen 2 (−548.58 kN) was 1.06 times that of specimen 1 (−517.15 kN). Although the positive yield drift of specimen 1 was slightly higher than that of specimen 2, their negative yield drifts were comparable. A notable disparity in load-bearing capacities between specimen 1 and specimen 2 was observed, with specimen 2 exhibiting a higher positive load-bearing capacity. This can be attributed to the enhanced compressive resistance of the infill wall and the increased frictional effect at the bottom contact surface during positive loading. Conversely, during the negative loading process, this frictional effect played a lesser role. At the peak point of the specimens, the positive peak load of specimen 2 (823.06 kN) was 1.03 times that of specimen 1 (802.05 kN), and the negative peak load of specimen 2 (−650.89 kN) was 1.07 times that of specimen 1 (−609.89 kN). The peak drifts of specimen 2 and specimen 1 were similar. The experimental results indicated that specimen 2, with two infill wall panels, exhibited superior overall performance compared to specimen 1, which incorporated a single integrated infill wall panel, demonstrating more comprehensive plastic damage development.

As shown in Table 3, during the initial stage of loading, specimen 1 and specimen 2 with the filled walls exhibited higher initial stiffness compared to specimen 3 [10]. Moreover, the skeletal load–displacement curves for each specimen demonstrated a consistent trend. As the loading drift increased, the positive peak loads of specimen 1 (802.05 kN) and specimen 2 (823.06 kN) were 269.13 kN and 290.14 kN greater than the positive peak load of specimen 3 (532.92 kN), respectively. During the positive loading process, the infill walls of specimen 1 and specimen 2 were subjected to compression, increasing the resisting overturning moment arm of the vertical force. After deducting the increased overturning moment, i.e., (480 kN × 0.8 m)/3.05 m = 125.9 kN, the positive peak loads of specimen 1 and specimen 2 were 676.15 kN and 697.16 kN, respectively. Compared with the positive peak load of specimen 3 (532.92 kN), it could be concluded that the infill walls of specimen 1 and specimen 2 contributed 143.13 kN and 164.14 kN, respectively, due to compression and contact surface friction. During the negative loading process, the four vertical bars in the edge column of the infill walls in specimen 1 and specimen 2 yielded, bearing a load of 138.16 kN. The negative yield loads of specimen 1 (−609.89 kN) and specimen 2 (−650.89 kN) were 136.97 kN and 95.97 kN greater than the negative yield load of specimen 3 (−513.92 kN), respectively. It was evident that during the negative loading process, the edge columns of specimen 1 and specimen 2 contributed the majority of the increased load-bearing capacity. Therefore, it can be observed that the infill walls made a limited contribution to the overall load-bearing capacity of the structures.

### 3.4. Structural Ductility

Ductility refers to the ability of a structure to undergo significant plastic deformation after reaching its elastic limit without experiencing significant strength and stiffness deterioration. The ductility coefficient is used to quantify the level of structural ductility, where the ductility coefficient represents the ratio of deformation at failure to that at yielding. In seismic design, ductility is a critical seismic design parameter. The formula for calculating the ductility coefficient is as follows:

(1) $$\mu =\frac{\delta_{u}}{\delta_{y}}$$

where *δ_y_* denotes the lateral drift at yielding and *δ_u_* denotes the lateral drift at failure (*F_u_* = 0.85*F_p_*).

Table 4 shows the structural ductility coefficients of the specimens. The ductility coefficient of specimen 2 was slightly higher than that of specimen 1. It was indicated that specimen 2, assembled with two pieces of infill wall panels, exhibited better ductility compared to specimen 1, which was assembled with a single infill wall panel. The ductility coefficients of all specimens were greater than 1.5, indicating good ductility and meeting the seismic performance requirements [35]. This suggested the reliability of the connection details in each specimen, surpassing the specified limit drift of 1/120 according to the seismic design code [35].

### 3.5. Secant Stiffness

The stiffness degradation of the specimens was described in this study by the slope of the lines connecting the peak points of each loading level in the hysteresis curve. The secant stiffness at each load level is defined as follows:

(2) $$K_{i}=\frac{\left|+F_{i}\right|+\left|-F_{i}\right|}{\left|+X_{i}\right|+\left|-X_{i}\right|}$$

where *F_i_* and *X_i_* are the peak load and the corresponding displacement in the first cycle of the *i*-th loading amplitude, respectively.

**Figure 9:**
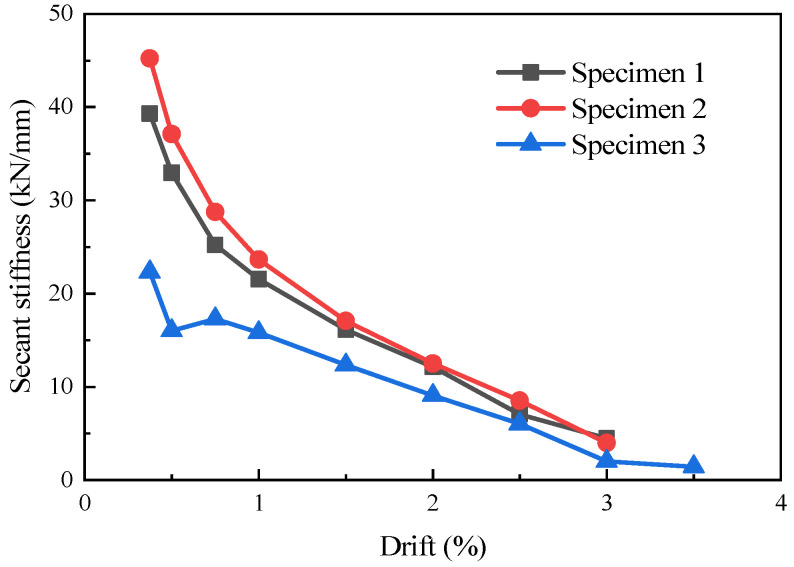
Stiffness degradation curves of the specimens [10].

The stiffness degradation curves of the specimens are shown in Figure 9. The initial secant stiffnesses of specimens 1, 2, and 3 were 39.29 kN/mm, 45.23 kN/mm, and 22.33 kN/mm, respectively. Specimen 2 exhibited the highest initial stiffness. This indicated that the infill wall enhanced the stiffness of the specimens. As loading progressed, specimens 1 and 2 experienced a faster rate of stiffness degradation compared to specimen 3. When subjected to a lateral drift of 2%, the secant stiffness degradation of specimens 1, 2, and 3 was 68.19%, 62.23%, and 59.42%, respectively. This was attributed to the more extensive cracking in specimens 1 and 2 compared to specimen 3. Considering the trend of stiffness degradation throughout the entire loading process, specimen 2 exhibited slightly better overall integrity during the early stages of loading, with higher stiffness. In the later stages of loading, the degradation curves of specimens 1 and 2 tended to align.

### 3.6. Energy Dissipation

**Figure 10:**
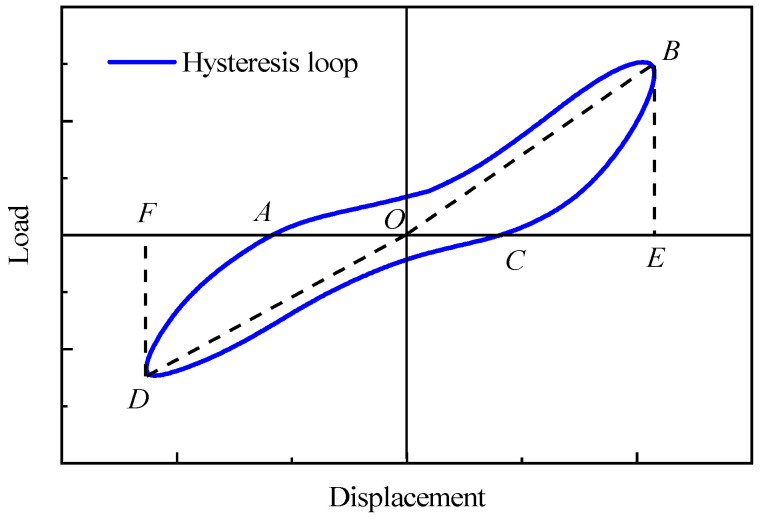
Schematic diagram for calculating equivalent viscous damping coefficient.

The equivalent viscous damping coefficient *ζ_eq_* employed in this study served as an indicator for assessing the energy dissipation of structures. The calculation of the equivalent viscous damping coefficient *ζ_eq_* was performed according to Equation (3).

(3) $$\zeta_{eq}=\frac{1}{2\pi }\cdot \frac{S_{ABCD}}{S_{\Delta BOE}+S_{\Delta DOF}}$$

where *S_ABCD_* represents the area enclosed by the hysteresis loop of one loading cycle, signifying the energy dissipated within a single cycle. *S*_Δ*BOE*_ and *S*_Δ*DOF*_ denote the triangular areas under the hysteresis loop in this cycle, as illustrated in Figure 10 for computational guidance.

**Figure 11:**
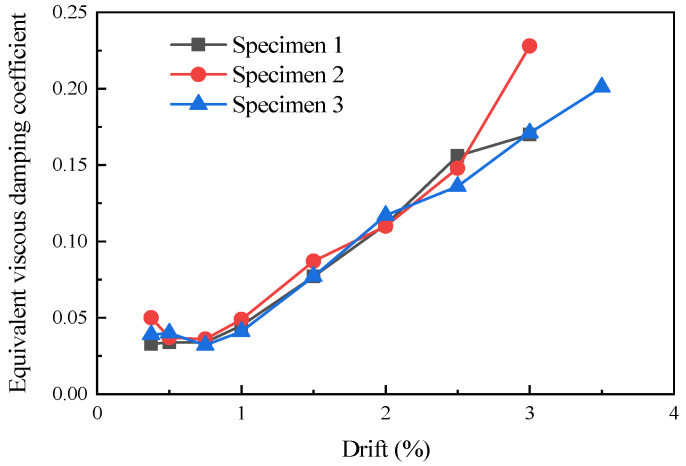
Equivalent viscous damping coefficient of the specimens [10].

Figure 11 shows the equivalent viscous damping coefficient of the specimens. The initial equivalent viscous damping coefficients of specimen 1 and specimen 2 were 0.03 and 0.05, respectively. When loaded to a lateral drift of 0.75%, the lower part of the concrete in specimen 1 began to crack, and the steel cage and angle steel connector worked together to dissipate energy, causing a rapid increase in the equivalent viscous damping coefficient. When loaded to a lateral drift of 2.5%, the cracks in specimen 1 had fully developed, and the main failure location of the concrete had already collapsed and spalled, causing the energy dissipation rate to begin to slow down. In contrast, after the full development of cracks in the shear wall of specimen 2, the infill wall continued to deteriorate, leading to a continuous increase in energy dissipation until the end of the loading process. At the end of the loading process, the equivalent viscous damping coefficient of specimen 2 was 35.3% higher than that of specimen 1. The results indicated that specimen 2, which was equipped with two infill wall panels, possessed a stronger energy dissipation capacity compared to specimen 1, which only had a single infill wall panel. This suggested that specimen 2 exhibited superior overall structural integrity.

### 3.7. Shear Deformation

**Figure 12:**
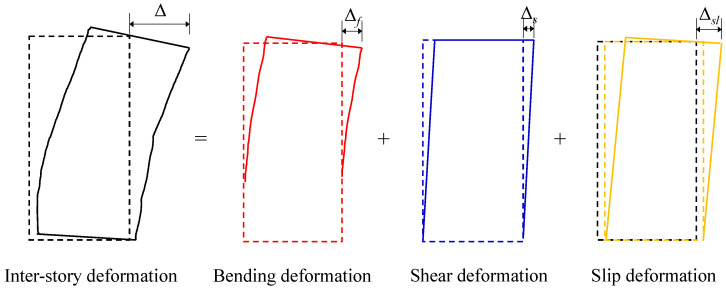
Inter-story deformation of the precast shear wall.

**Figure 13:**
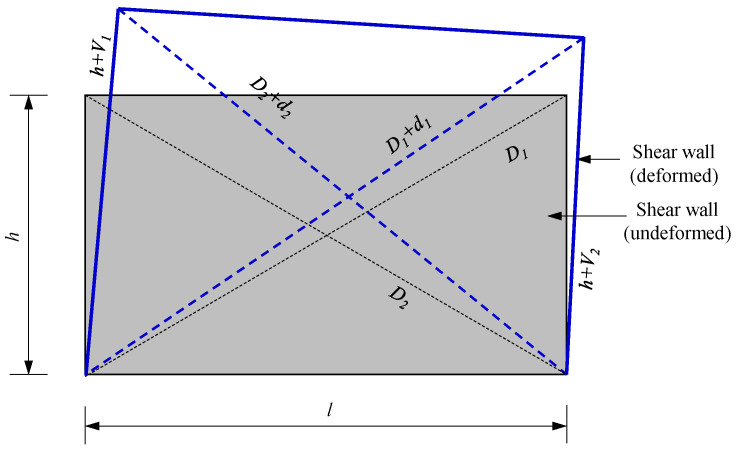
Schematic diagram for shear deformation calculation.

Under the action of horizontal lateral force, the inter-story deformation (Δ) of the precast shear wall consists of three components: bending deformation (Δ*_f_*), shear deformation (Δ*_s_*), and slip deformation (Δ*_sl_*), as illustrated in Figure 12. Bending deformation occurs when there is no relative slip, and it is the vertical bending rotation deformation of the wall. In this study, bending deformation is calculated as Δ*_f_* = 1 − Δ*_s_*. Slip deformation is the deformation generated by relative slip at the horizontal connection of the precast shear wall. Since the slip value in this study was extremely small, it can be neglected. Shear deformation is the deformation produced by shear stress on the wall in the absence of relative slip. The calculation procedure for shear deformation is illustrated in Figure 13. In this study, shear deformation was calculated using Equation (4).

(4) $$\Delta_{s}=\frac{1}{2}\left[\sqrt{{\left(D_{1}+d_{1}\right)}^{2}-h^{2}}-\sqrt{{\left(D_{2}+d_{2}\right)}^{2}-h^{2}}\right]$$

**Figure 14:**
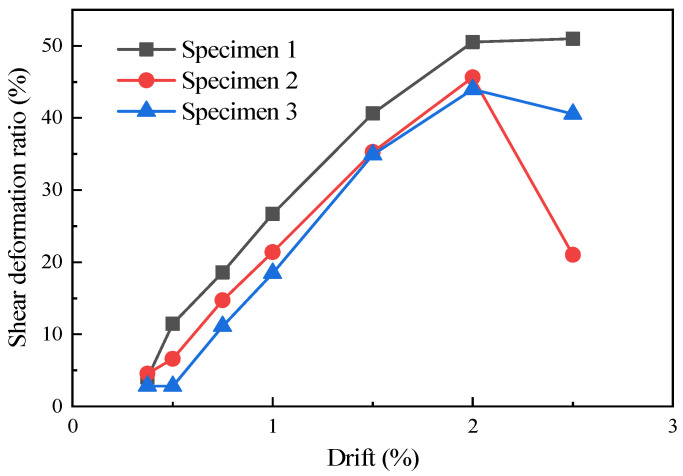
Shear deformation ratio of the specimens [10].

Based on the calculated shear deformations, the shear deformation ratios of the specimens were determined, as shown in Figure 14. It could be observed that specimens with infill walls exhibited higher shear deformation ratios compared to those without infill walls. Furthermore, specimen 1, which was furnished with a single infill wall panel, demonstrated a higher shear deformation ratio compared to specimen 2, which featured two infill wall panels.

Steel connectors have been previously proposed in the literature [39,40,41]. However, the construction and function of steel connectors in their proposed structure differ from the role of angle steel connectors in the structure presented in this paper. Fan et al. [39] introduced a steel plate wall with stiffeners and a concrete slab in the box-plate steel structure, where the stiffened steel plates were directly used as load-carrying walls and floors. Mi et al. [40] proposed a steel-reinforced precast concrete shear wall with replaceable low-yield-point steel energy dissipators. In their design, two angle steels are diagonally arranged within the precast wall to enhance structural performance. The research objective focused on the seismic performance of structures when reinforced walls were combined with additional energy dissipation devices, rather than emphasizing the performance of angle steel connections. Yang et al. [41] proposed a composite structure using engineered cementitious composites and steel, with angle steel embedded into the wall and connected to surrounding edge components through bolts. This study centers on the precast concrete shear wall structure. In practical engineering, precast concrete shear walls often feature construction joints, which can significantly impact the seismic performance of the structure, particularly under axial tension. Angle steel connectors were used to improve the shear resistance of the horizontal joints between the precast reinforced concrete shear wall and the foundation, as well as to provide temporary support for specimen positioning and installation. The research team has previously conducted a comprehensive investigation into the seismic performance of this new precast shear wall system with an angle steel connector under axial tension and compression [10,30]. The findings indicated that this new precast shear wall system with an angle steel connector demonstrated superior seismic performance compared to traditional lap-jointed prefabricated concrete shear walls, grouting sleeve-connected prefabricated concrete shear walls, and monolithic cast-in-place shear walls. The current study conducted experimental research on the placement and construction of infill walls within this new precast shear wall system and examined their effects on the seismic performance of the structure. The goal is to provide valuable references for the application and design of related structures while maintaining the reliability of this new precast shear wall system with an angle steel connector.

## 4. Conclusions

This paper presented an experimental study on the seismic behavior of a new precast shear wall system with different infill wall constructions. The following conclusions were drawn:

(1) Specimen 1 exhibited fewer cracks in the infill wall section, with notable cracks observed mainly in the edge columns. Additionally, specimen 2 displayed a more extensive development of cracks within the shear wall section compared to specimen 1. The specimen with two infill wall panels performed better in terms of overall performance compared to the one with a single continuous infill wall panel.
(2) During the negative loading process, the edge columns of specimen 1 and specimen 2 contributed the majority of the increased load-bearing capacity. Meanwhile, the infill walls made a limited contribution to the overall load-bearing capacity of the structures.
(3) Specimen 2, assembled with two pieces of infill wall panels, exhibited better ductility compared to specimen 1, which was assembled with a single infill wall panel. The ductility coefficients of all specimens were greater than 1.5, indicating good ductility and meeting the seismic performance requirements.
(4) Specimen 2, which was equipped with two infill wall panels, possessed a stronger energy dissipation capacity compared to specimen 1, which only had a single infill wall panel. This suggested that specimen 2 exhibited superior overall structural integrity.

## Figures and Tables

**Table 1:** Measured average mechanical properties of the concrete (unit: MPa).

No.	Post-Casting Part	Cast-In-Suit Part
1	49.33	48.46
2	48.14	46.93
3	49.52	48.44

**Table 2:** Measured average mechanical properties of the steel (unit: MPa or %).

Diameter, *D*	Grade	Yield Strength, *f_y_*	Ultimate Strength, *f_u_*	Elongation, *δ*
8	HRB400	436.7	614.8	25.7
10		449.1	631.4	25.1
12		442.4	604.4	24.6
-	Q235	239.6	414.7	20.8

**Table 3:** Characteristic values of load-bearing capacity of the specimens (unit: kN or %).

No.	Loading Direction	Yield Load, *F_y_*	Yield Drift, *δ_y_*	Peak Load, *F_p_*	Peak Drift, *δ_p_*
Specimen 1	Positive	662.13	1.03	802.05	1.75
	Negative	−517.15	−0.94	−609.89	−1.66
Specimen 2	Positive	677.98	0.76	823.06	1.72
	Negative	−548.58	−0.95	−650.89	−1.67
Specimen 3 [10]	Positive	502.55	1.26	532.92	1.66
	Negative	−430.63	−0.74	−513.92	−1.61

**Table 4:** Structural ductility coefficients of the specimens.

No.	Loading Direction	Yield Drift, *δ_y_* (%)	Failure Drift, *δ_u_* (%)	Ductility Coefficient, *μ*
Specimen 1	Positive	1.03	2.27	2.21
	Negative	−0.94	−2.35	2.50
Specimen 2	Positive	0.76	2.45	3.20
	Negative	−0.95	−2.57	2.71
Specimen 3 [10]	Positive	1.26	2.45	1.94
	Negative	−0.74	−2.42	3.27

## Data Availability

Data available on request due to restrictions eg privacy or ethical. The data presented in this study are available on request from the corresponding author. The data are notpublicly available due to follow-up research on the work.
